# High-Throughput Phenotyping of the Symptoms of Alzheimer Disease and Related Dementias Using Large Language Models: Cross-Sectional Study

**DOI:** 10.2196/66926

**Published:** 2025-06-03

**Authors:** You Cheng, Mrunal Malekar, Yingnan He, Apoorva Bommareddy, Colin Magdamo, Arjun Singh, Brandon Westover, Shibani S Mukerji, John Dickson, Sudeshna Das

**Affiliations:** 1 Department of Neurology Massachusetts General Hospital Cambridge, MA United States; 2 Harvard Medical School Boston, MA United States; 3 Department of Neurology Beth Israel Hospital Boston Boston, MA United States

**Keywords:** electronic health record, Alzheimer disease and related dementias, large language model, disease phenotyping, symptom extraction, differential diagnosis, brain volume

## Abstract

**Background:**

Alzheimer disease and related dementias (ADRD) are complex disorders with overlapping symptoms and pathologies. Comprehensive records of symptoms in electronic health records (EHRs) are critical for not only reaching an accurate diagnosis but also supporting ongoing research studies and clinical trials. However, these symptoms are frequently obscured within unstructured clinical notes in EHRs, making manual extraction both time-consuming and labor-intensive.

**Objective:**

We aimed to automate symptom extraction from the clinical notes of patients with ADRD using fine-tuned large language models (LLMs), compare its performance to regular expression-based symptom recognition, and validate the results using brain magnetic resonance imaging (MRI) data.

**Methods:**

We fine-tuned LLMs to extract ADRD symptoms across the following 7 domains: memory, executive function, motor, language, visuospatial, neuropsychiatric, and sleep. We assessed the algorithm’s performance by calculating the area under the receiver operating characteristic curve (AUROC) for each domain. The extracted symptoms were then validated in two analyses: (1) predicting ADRD diagnosis using the counts of extracted symptoms and (2) examining the association between ADRD symptoms and MRI-derived brain volumes.

**Results:**

Symptom extraction across the 7 domains achieved high accuracy with AUROCs ranging from 0.97 to 0.99. Using the counts of extracted symptoms to predict ADRD diagnosis yielded an AUROC of 0.83 (95% CI 0.77-0.89). Symptom associations with brain volumes revealed that a smaller hippocampal volume was linked to memory impairments (odds ratio 0.62, 95% CI 0.46-0.84; *P*=.006), and reduced pallidum size was associated with motor impairments (odds ratio 0.73, 95% CI 0.58-0.90; *P*=.04).

**Conclusions:**

These results highlight the accuracy and reliability of our high-throughput ADRD phenotyping algorithm. By enabling automated symptom extraction, our approach has the potential to assist with differential diagnosis, as well as facilitate clinical trials and research studies of dementia.

## Introduction

Alzheimer disease and related dementias (ADRD) encompass a group of disorders characterized by cognitive and behavioral impairments, which progressively affect memory, thinking, and activities of daily living [1]. Among them, Alzheimer disease (AD) is the most common form of dementia and affects approximately 6.7 million individuals in the United States [1]. Other major types of ADRD include dementia with Lewy bodies (DLB), frontotemporal dementia (FTD; behavioral variant), Parkinson disease (PD), primary progressive aphasia (PPA), and vascular cognitive impairment (VCI), each presenting unique symptom profiles with overlapping characteristics. For example, AD typically presents with memory loss [2]; DLB with visual hallucinations, motor symptoms, and sleep disturbances [3]; FTD with behavioral and language symptoms [4]; and PD with motor symptoms [5]. However, clinical presentations and symptoms vary with neuropathology, which contributes to diagnostic challenges. Documentation of ADRD symptoms often exists solely within unstructured clinical notes in electronic health records (EHRs) without any standardization, and manual chart review is error prone and time consuming. The development of an artificial intelligence algorithm for automatic symptom extraction from clinical notes could significantly aid in overcoming these challenges, thereby offering substantial benefits for diagnosis and intervention strategies. Additionally, the symptom data in clinical notes have the potential to facilitate research studies, for example, studies of the longitudinal progression of symptoms in patients with ADRD or how symptoms are documented, shedding light on both medical patterns and recording practices [6].

Symptom extraction is often performed by manual expert chart review, which is inefficient and labor intensive. Traditional text mining and natural language processing (NLP) techniques, which rely on symptom-related keywords specified by domain experts [7,8], can facilitate the symptom extraction process. For example, Vijayakrishnan et al [9] developed a rule-based NLP pipeline to identify heart failure symptoms using the Framingham heart failure diagnostic criteria. Jackson et al [10] created a unified NLP model for extracting severe mental illness symptoms based on a keyword lexicon crafted by psychiatrists. Moreover, Forsyth et al [11] developed a machine learning model to extract breast cancer symptoms based on a code book developed by physicians. However, these rule-based or keyword-dependent methods are still susceptible to missing semantic relationships and contextual information.

In contrast to traditional NLP techniques, the advent of deep learning–based large language transformer models [12-14] presents a significant improvement by understanding contextual information and semantic relationships in clinical notes. In particular, large language models (LLMs) are adept at recognizing complex patterns and relationships within texts using an attention-based transformer model [15]. For example, a recent study used LLMs to extract cannabis use and documentation in EHRs among children and young adults [16]. In another study, researchers created an LLM-based symptom extraction model that can be applied to extract COVID-19 symptoms from Twitter data [17]. Indeed, by understanding the context of keywords and terminologies, these models can enable more accurate and sensitive symptom extraction.

In this study, we used LLMs [12,13,18] to extract symptoms from the clinical notes of patients diagnosed with ADRD. Symptoms were categorized into 7 domains: *memory*, *executive function*, *motor*, *language*, *visuospatial*, *neuropsychiatric*, and *sleep*, with distinction as impaired, intact, or no information. This method quantified symptom occurrences for further analysis. The overall aim was to develop an effective model for automated symptom extraction, which may not only facilitate the differential diagnosis of ADRD (AD, DLB, FTD, PD, PPA, and VCI), but also support research on heterogeneity within these subtypes. To evaluate the effectiveness of our LLM-based approach, we compared it against a traditional rule-based method using regular expressions for symptom extraction. We further validated the model’s symptom predictions using brain volume data derived from magnetic resonance imaging (MRI).

## Methods

### Study Dataset

The dataset consisted of the EHR data of patients from the Massachusetts General Hospital (MGH) memory clinic (collected between 2015 and 2022), who were over 50 years old at their first visit and had at least two MGH memory clinic encounters. The dataset was further filtered to exclude patients without an office or telemedicine visit or those who did not have a progress note with at least 512 characters. The final dataset was filtered to only include patients with 1 of 6 ADRD diagnoses during their latest encounter: AD, DLB, FTD, PD, PPA, or VCI, and without mixed dementia in their EHR history. See Multimedia Appendix 1 for the full list of diagnosis names by ADRD category.

### Ethical Considerations

This study was approved by the Mass General Brigham Institutional Review Board (protocol 2015P001915), with a waiver of informed consent granted for secondary analysis of electronic health records. No participant compensation was provided. Data were extracted from Epic and securely stored on servers within the Mass General Brigham firewall, with access limited to authorized study personnel in accordance with institutional privacy and data security policies.

### Preprocessing

To process the notes, we applied *medspaCy*, a specialized text analysis tool for clinical notes [19]. We extracted key sections of the notes that held important information regarding the patient’s symptoms such as medical history, examination, and impression. The extraction tool was customized for each physician’s template. Subsequently, we sampled notes based on ADRD diagnoses and split notes into sentences or phrases for symptom annotation.

### Annotation

**Figure 1:**
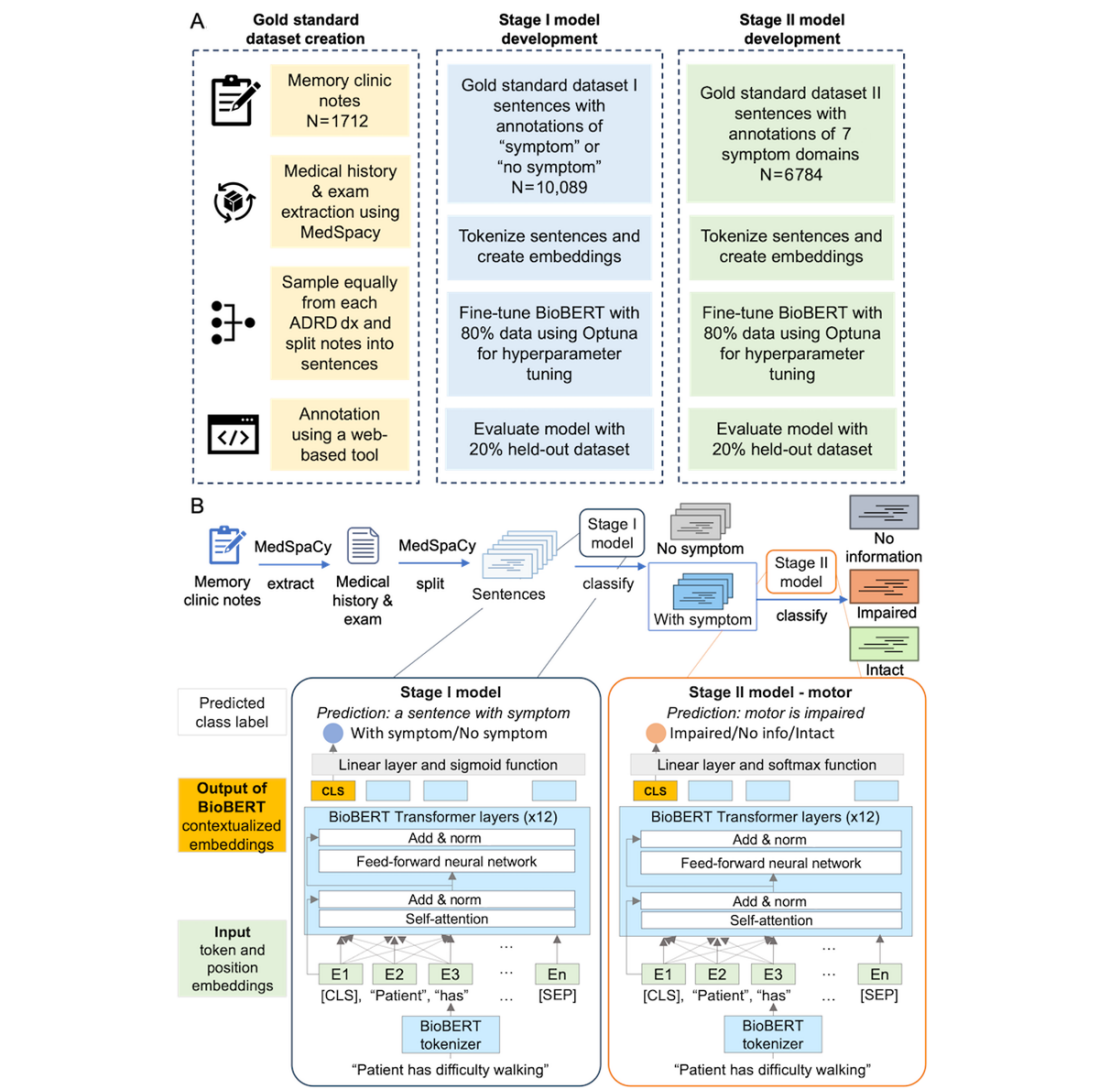
Model development and architecture. (A) Gold standard dataset creation and model development. This workflow describes the development of a 2-tier hierarchical model to classify symptoms in clinical notes. Initially, 1712 memory clinic notes are processed, and sentences sampled across various Alzheimer disease and related dementias (ADRD) diagnoses are manually annotated using a web tool, producing 2 gold standard datasets: one identifying symptom presence, and another categorizing symptom status across 7 domains. The 2 classification models, built on BioBERT, undergo fine-tuning using 80% of the data and testing using 20% of a held-out dataset. (B) Illustration of the application of BioBERT in stage I and stage II models for symptom extraction. dx: diagnosis.

An expert (AB) conducted thorough review of the medical literature and identified symptoms from seven domains typically present in patients living with ADRD: (1) memory, (2) executive function, (3) motor, (4) language, (5) visuospatial, (6) neuropsychiatric (which also incorporates symptoms related to behavior and mood), and (7) sleep (Multimedia Appendix 2). A behavioral neurologist (JD) provided critical input throughout both processes. Subsequently, another expert (MM) annotated sentences or phrases as *symptom* (patient shows intact or impaired symptoms) or *no symptom* (no information on patient symptoms). Further, MM annotated sentences or phrases as *intact*, *impaired*, or *no information* for each of the 7 symptom domains, using a web-based JavaScript annotation tool developed by AS. Using these annotations, we created 2 gold standard datasets: *gold standard dataset I* (composed of sentences or phrases labeled as *symptom* or *no symptom*) and *gold standard dataset II* (composed of sentences or phrases labeled as *intact*, *impaired*, or *no information* across the 7 symptom domains). The process for creating the gold standard dataset is illustrated in Figure 1A.

### Symptom Recognition Using BioBERT

We developed a 2-tier hierarchical model for symptom extraction. The *stage I binary symptom classification model* classified each input sentence as *symptom* or *no symptom*. The *stage II multi-label symptom classification model* is composed of 7 distinct models, with each trained to classify sentences or phrases from 1 of the 7 symptom domains, namely *memory*, *executive function*, *motor*, *language*, *visuospatial*, *neuropsychiatric*, and *sleep*. Each *stage II multi-label symptom classification model* classifies sentences or phrases into 3 categories: *impaired*, *intact*, and *no information*. The *impaired* category encapsulates symptoms indicative of impairment within the specific domain, highlighting manifestations of dysfunction. Conversely, the *intact* category encompasses symptoms that reflect normal functioning of the respective symptom domain. The *no information* category encompasses all remaining symptoms from other categories (eg, a sentence that only mentions *motor* symptom is categorized as *no information* in the *memory* model), supplemented by nonsymptomatic sentences.

Both the *stage I binary symptom classification model* and *stage II multi-label symptom classification model* were developed using *BioBERT* [20], an LLM pretrained on a large corpus of biomedical text (eg, PubMed abstracts and PubMed Central full-text articles) and implemented using the HuggingFace’s *Python* *transformers* package (version 4.8.2) [21]. The *stage I binary symptom classification model* was initialized with its pretrained parameters of BioBERT and then fine-tuned on the *gold standard dataset I* (80% training set, 20% hold-out set)*.* The *stage II multi-label symptom classification model* was again initialized with pretrained parameters and later fine-tuned on *gold standard dataset II* (80% training set, 20% hold-out set). *Optuna* hyperparameter tuning was used to tune the hyperparameters for both models, including training epochs, batch size, and learning rate, with a 20-trial study to maximize the area under the precision-recall curve. An early stopping criterion was implemented to cease training if the loss did not change substantially in 4 epochs, preventing overfitting.

Figure 1B shows how we used BioBERT for the stage I and stage II models. We used the pretrained BioBERT model as a starting point and fine-tuned it for our task. As shown in Figure 1B, the extracted sentences are first processed through the BioBERT tokenizer, which splits the raw text into tokens. For example, the sentence “Patient has difficulty walking” is tokenized. Then, each token is converted into a pretrained embedding, capturing the semantic meaning of the word in the context of the sentence, along with a position embedding that encodes the token’s location within the sequence to help the model understand word order and structure. A [CLS] token is added at the beginning of each sentence. Its embedding is used to represent the aggregated meaning of the entire sentence. A [SEP] token is placed at the end to signify the boundary between input tokens. E (embedding) from 1 to n represents the token embeddings, with the total count of n including [CLS] and [SEP]. These embeddings are passed through BioBERT’s transformer layers, which use self-attention and feed-forward neural networks to generate context-aware embeddings. As the sentence passes through the layers, the embedding of the [CLS] token becomes enriched with contextualized information derived from the full sentence, which represents the overall meaning of the input. Finally, the embedding of the [CLS] token is used as the input for the linear layer, which calculates the logits for each class. Sigmoid (for binary classification) or SoftMax (for multi-class classification) as a decision function is applied to these logits to obtain class probabilities, and the class with the highest probability is selected as the model’s predicted label. We fine-tuned BioBERT separately for stage I (binary classification) using gold standard dataset I and for stage II (multi-label classification) using gold standard dataset II. The fine-tuning process primarily involves adjusting the parameters of the BioBERT transformer layers and the linear layer to optimize performance for each stage’s specific classification task.

We also experimented with other pretrained models as part of our preliminary experiments, including ClinicalBERT, RoBERTa, and LLaMA 2, with the latter being a generative transformer model. Despite fine-tuning (for ClinicalBERT and RoBERTa) or prompt engineering (for LLaMA 2), the models did not achieve the same level of performance as BioBERT in symptom classification based on the area under the receiver operating characteristic curve (AUROC) and *F*_1_-score. All text processing and LLM development procedures were conducted in *Python* (version 3.8.15).

### Symptom Recognition Using Regular Expressions

We created a list of regex patterns for ADRD symptoms to compare the efficacy of our advanced LLM approach with the traditional rule-based regex technique. First, 100 patient visit notes across the 6 ADRD diagnoses (AD, DLB, FTD, PD, PPA, and VCI) were randomly sampled. These notes were analyzed to identify examples from each of the 7 symptom domains (*memory*, *executive function*, *motor*, *language*, *visuospatial*, *neuropsychiatric*, and *sleep*) and develop a comprehensive set of regex patterns for each symptom domain. An expert behavioral neurologist (JD) provided critical guidance throughout this process. Next, these regex patterns were used to flag sentences or phrases corresponding to each symptom domain in the entire set of visit notes. The symptom counts for each note were then aggregated to calculate the total number of matches for each domain. For the full list of regex patterns, please see Multimedia Appendix 3.

### Validation via ADRD Differential Diagnosis

We compiled symptom counts across 7 domains (*memory*, *executive function*, *motor*, *language*, *visuospatial*, *neuropsychiatric*, and *sleep*) based on predictions of our 2-tier hierarchical model on the entire set of visit notes. These symptom counts served as input features for a multinomial L1-regularized logistic regression model to classify 6 ADRD diagnoses (AD, DLB, FTD, PD, PPA, and VCI). To optimize the model, we employed 5-fold cross-validation and grid search cross-validation to determine the optimal value of alpha for L1 regularization using the *Python* *scikit-learn* (version 0.24.2) package. Additionally, we incorporated the aggregated symptom counts, derived from applying the ADRD symptom regex patterns (Multimedia Appendix 3) on the same dataset, as features in the machine learning model. We hypothesized that symptoms identified with our 2-tier hierarchical model would have superior performance than those derived from regex patterns in predicting ADRD diagnoses. All ADRD differential diagnosis analyses were conducted in *Python* (version 3.8.15).

### Validation via MRI Brain Volume Data

To evaluate symptom predictions using MRI, we selected memory clinic notes with an MRI scan performed within 1 year of the visit. We ensured that none of these notes overlapped with the gold standard datasets. Each clinical note was matched with a unique MRI scan from the Mass General Brigham patient database, with the imaging date being within 1 year of the visit date. The *SynthSeg+* pipeline [22] was used for brain segmentation and volume estimation. Only those images whose subcortical regions collectively surpassed a threshold of 0.65 in the average automated quality control score were selected for further analysis. For patients with multiple eligible clinical images, the final brain volume was determined by averaging the volumes across all qualifying images. Furthermore, to account for individual differences, the volume of each brain region was normalized by the intracranial volume.

In our brain volume analysis, we first selected *a priori* brain regions associated with 2 of the most commonly disrupted functions in patients with ADRD: *memory* and *motor*. For *memory* symptoms, we investigated the bilateral hippocampus and entorhinal cortex, both associated with the memory of recent events, as well as the prefrontal cortex, which is related to immediate memory [2,23-25]. For *motor* symptoms, our evaluation encompassed the bilateral primary motor cortex, the secondary motor cortex, the basal ganglia (including the caudate, putamen, pallidum, and nucleus accumbens) along with the thalamus (a structure with strong connections to the basal ganglia), and the cerebellar gray and white matter [26-29].

Logistic regression was used to evaluate the volumes of brain regions associated with symptoms, with a contrast of cases having *impaired* symptoms and those having either *intact* symptoms or *no information*. The analysis was conducted for both *memory* and *motor* symptoms, with adjustments made for age and sex, using the function *glm* in the *R stats* (version 4.3.2) package. The reported results were adjusted for multiple comparisons using the Benjamini-Hochberg method [30]. All MRI brain volume analyses were conducted in *R* (version 4.2.1; R Core Team). For a detailed workflow of validation using MRI, see Figure S1 in Multimedia Appendix 4.

## Results

### Study Data

**Figure 2:**
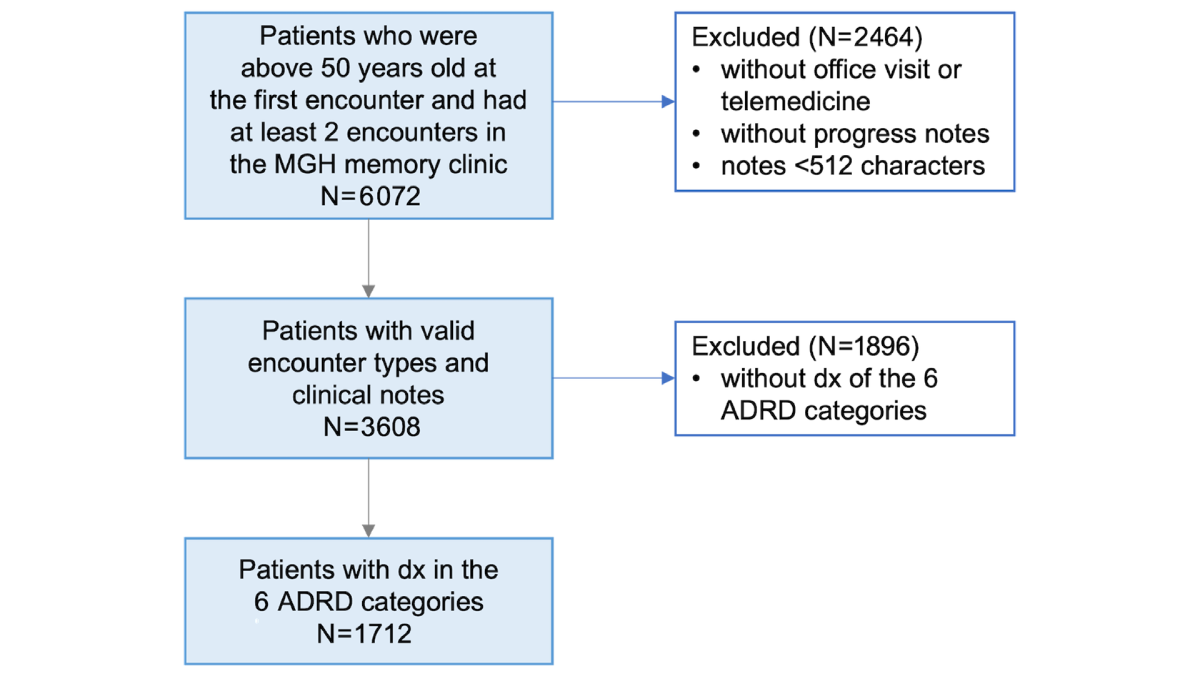
Consort diagram of the selection of patients with Alzheimer disease and related dementias (ADRD). This consort diagram illustrates the patient selection process from the Massachusetts General Hospital (MGH) memory clinic. dx: diagnosis.

The study data consisted of visit notes from the latest encounters of 1712 patients (Figure 2). The visit notes were from 866 (50.6%) male and 846 (49.4%) female patients, with an average age at visit of 77.5 (SD 8.3) years. All patients had 1 of the following ADRD diagnoses: AD, DLB, FTD, PD, PPA, and VCI. The patient demographics are described in Table 1.

From these 1712 visit notes, we compiled 2 gold standard datasets. Gold standard dataset I included 10,089 sentences or phrases labeled as *symptom* (n=5468, 54.2%) or *no symptom* (n=4621, 45.8%). Gold standard dataset II included 6784 sentences or phrases labeled as *intact*, *impaired*, or *no information* across the 7 symptom domains. The ADRD diagnoses in dataset II predominantly included AD (2862/6784, 42.2%) and DLB (1866/6784, 27.5%), followed by FTD (879/6784, 13.0%), PD (628/6784, 9.3%), VCI (479/6784, 7.1%), and PPA (70/6784, 1.0%). Specifically, AD had the highest counts for *memory* and *visuospatial* symptoms; DLB led in *executive function* symptoms; PD was predominant in *motor* symptoms; PPA led in *language* symptoms; and FTD was notable for *neuropsychiatric* and *sleep* symptoms, with high counts also noted in *visuospatial* and *sleep* symptoms for VCI and DLB, respectively (refer to Table 2 for detailed distributions). A standardized mean difference (SMD) threshold of 0.1 was employed to assess the equilibrium of each metric, with measurements exceeding 0.1 indicating a comparative lack of balance. The MRI validation dataset included 582 visit notes from 528 unique patients and had clinical MRI performed within 1 year (Figure S2 in Multimedia Appendix 4). For demographic distribution related to these visit notes, refer to the last column of Table 1.

**Table 1:** Summary statistics of the demographic and clinical characteristics of 1712 patients, including a subset of 582 visits from 528 patients with valid magnetic resonance imaging data.

Characteristic		Total sample (N=1712)	MRI^a^ sample (n=582)
Age at visit (years), mean (SD)		77.5 (8.3)	76.3 (7.3)
**Sex, n (%)**			
	Female	846 (49.4)	279 (47.9)
	Male	866 (50.6)	303 (52.1)
**Race and ethnicity, n (%)**			
	Non-Hispanic White	1317 (76.9)	459 (78.9)
	Non-Hispanic Black	40 (2.0)	10 (1.7)
	Non-Hispanic Asian	42 (2.5)	16 (2.7)
	Hispanic or Latino	54 (3.2)	20 (3.4)
	American Indian or Alaska Native	3 (0.2)	1 (0.2)
	Other	25 (1.5)	9 (1.5)
	Unavailable	231 (13.5)	67 (11.5)
**Visit diagnosis, n (%)**			
	Alzheimer disease	1117 (65.2)	378 (64.9)
	Dementia with Lewy bodies	143 (8.4)	44 (7.6)
	Frontotemporal dementia	195 (11.4)	67 (11.5)
	Parkinson disease	53 (3.1)	15 (2.6)
	Primary progressive aphasia	89 (5.2)	24 (4.1)
	Vascular cognitive impairment	115 (6.7)	54 (9.3)

^a^MRI: magnetic resonance imaging.

**Table 2:** Summary statistics of gold standard dataset II.

Characteristic			Total (N=6784)		AD^a^ (n=2862)		DLB^b^ (n=1866)		FTD^c^ (n=879)		PD^d^ (n=628)		PPA^e^ (n=70)		VCI^f^ (n=479)		SMD^g^	
Age at visit (years), mean (SD)			77.7 (7.9)		79.9 (7.43)		74.8 (7.4)		75.8 (7.3)		75 (7.7)		72.4 (7.3)		83.6 (7.0)		0.661^h^	
**Sex, n (%)**																		0.540^h^
	Female	3221 (47.5)		1637 (57.2)		468 (25.1)		650 (73.9)		142 (22.6)		43 (61.4)		281 (58.7)				
	Male	3563 (52.5)		1225 (42.8)		1398 (74.9)		229 (26.1)		486 (77.4)		27 (38.6)		198 (41.3)				
**Race and ethnicity, n (%)**																		0.844^h^
	Non-Hispanic White	4613 (68.0)		1948 (68.1)		1498 (80.3)		251 (28.6)		485 (77.2)		68 (97.1)		363 (75.8)				
	Non-Hispanic Black	61 (0.9)		11 (0.4)		15 (0.8)		19 (2.2)		0 (0.0)		0 (0.0)		16 (3.3)				
	Non-Hispanic Asian	213 (3.1)		109 (3.8)		48 (2.6)		7 (0.8)		41 (6.5)		0 (0.0)		8 (1.7)				
	Hispanic or Latino	353 (5.2)		324 (11.3)		0 (0.0)		0 (0.0)		18 (2.9)		0 (0.0)		11 (2.3)				
	American Indian or Alaska Native	0 (0.0)		0 (0.0)		0 (0.0)		0 (0.0)		0 (0.0)		0 (0.0)		0 (0.0)				
	Other	64 (0.9)		64 (2.2)		0 (0.0)		0 (0.0)		0 (0.0)		0 (0.0)		0 (0.0)				
	Unavailable	1480 (21.8)		406 (14.2)		305 (16.3)		602 (68.5)		84 (13.4)		2 (2.9)		81 (16.9)				
**Memory, n (%)**																		0.275^h^
	Impaired	767 (11.3)		493 (17.2)		143 (7.7)		33 (3.8)		29 (4.6)		5 (7.1)		64 (13.4)				
	Intact	219 (3.2)		98 (3.4)		23 (1.2)		49 (5.6)		15 (2.4)		2 (2.9)		32 (6.7)				
	No information	5798 (85.5)		2271 (79.4)		1700 (91.1)		797 (90.7)		584 (93.0)		63 (90.0)		383 (80.0)				
**Executive function, n (%)**																		0.173^h^
	Impaired	797 (11.7)		371 (13.0)		256 (13.7)		43 (4.9)		68 (10.8)		5 (7.1)		54 (11.3)				
	Intact	240 (3.5)		118 (4.1)		70 (3.8)		13 (1.5)		16 (2.5)		2 (2.9)		21 (4.4)				
	No information	5747 (84.7)		2373 (82.9)		1540 (82.5)		823 (93.6)		544 (86.6)		63 (90.0)		404 (84.3)				
**Motor, n (%)**																		0.562^h^
	Impaired	1202 (17.7)		321 (11.2)		555 (29.7)		32 (3.6)		236 (37.6)		8 (11.4)		50 (10.4)				
	Intact	792 (11.7)		300 (10.5)		246 (13.2)		65 (7.4)		117 (18.6)		20 (28.6)		44 (9.2)				
	No information	4790 (70.6)		2241 (78.3)		1065 (57.1)		782 (89.0)		275 (43.8)		42 (60.0)		385 (80.4)				
**Language, n (%)**																		0.345^h^
	Impaired	545 (8.0)		214 (7.5)		89 (4.8)		167 (19.0)		31 (4.9)		19 (27.1)		25 (5.2)				
	Intact	263 (3.9)		104 (3.6)		54 (2.9)		54 (6.1)		22 (3.5)		5 (7.1)		24 (5.0)				
	No information	5976 (88.1)		2544 (88.9)		1723 (92.3)		658 (74.9)		575 (91.6)		46 (65.8)		430 (89.8)				
**Visuospatial, n (%)**																		0.154^h^
	Impaired	359 (5.3)		196 (6.8)		90 (4.8)		11 (1.3)		31 (4.9)		2 (2.9)		29 (6.1)				
	Intact	153 (2.3)		69 (2.4)		29 (1.6)		20 (2.3)		18 (2.9)		1 (1.4)		16 (3.3)				
	No information	6272 (92.5)		2597 (90.7)		1747 (93.6)		848 (96.5)		579 (92.2)		67 (95.7)		434 (90.6)				
**Neuropsychiatric, n (%)**																		0.453^h^
	Impaired	740 (10.9)		274 (9.6)		162 (8.7)		236 (26.8)		25 (4.0)		1 (1.4)		42 (8.8)				
	Intact	644 (9.5)		331 (11.6)		110 (5.9)		97 (11.0)		16 (2.5)		4 (5.7)		86 (18.0)				
	No information	5400 (79.6)		2257 (78.9)		1594 (85.4)		546 (62.1)		587 (93.5)		65 (92.9)		351 (73.3)				
**Sleep, n (%)**																		0.246^h^
	Impaired	333 (4.9)		98 (3.4)		125 (6.7)		76 (8.6)		25 (4.0)		0 (0.0)		9 (1.9)				
	Intact	157 (2.3)		74 (2.6)		41 (2.2)		16 (1.8)		9 (1.4)		0 (0.0)		17 (3.5)				
	No information	6294 (92.8)		2690 (94.0)		1700 (91.1)		787 (89.5)		594 (94.6)		70 (100.0)		453 (94.6)				

^a^AD: Alzheimer disease.

^b^DLB: dementia with Lewy bodies.

^c^FTD: frontotemporal dementia.

^d^PD: Parkinson disease.

^e^PPA: primary progressive aphasia.

^f^VCI: vascular cognitive impairment.

^g^SMD: standardized mean difference.

^h^Indicates comparative lack of balance.

### Symptom Recognition Using a Transformer-Based Language Model

We trained, validated, and tested a transformer-based LLM to identify symptoms related to ADRD diagnoses. The symptom extraction process was executed through a 2-stage framework. The stage I binary symptom classification model categorized sentences as either *symptom* or *no symptom*. The model attained a micro-averaged AUROC of 1.00 (95% CI 0.99-1.00), along with a micro-averaged *F*_1_-score of 0.98 (95% CI 0.97-0.98), micro-averaged precision of 0.98 (95% CI 0.97-0.98), and micro-averaged recall of 0.98 (95% CI 0.97-0.98), highlighting its ability to accurately detect symptom presence. The 95% CIs for each metric reflect the reliability of these estimates, confirming the model’s overall efficacy in symptom classification across diverse clinical features.

This initial classification is followed by the use of the stage II multi-label symptom classification models, which further classify each detected symptom into *impaired*, *intact*, and *no information*. The 7 stage II models are tailored to each specific domain (*memory*, *executive function*, *motor*, *language*, *visuospatial*, *neuropsychiatric*, and *sleep*). All symptom domains showed robust model performance, with micro-averaged AUROC values of 0.97-0.99, micro-averaged *F*_1_-score values of 0.89-0.96, micro-averaged precision values of 0.87-0.96, and micro-averaged recall values of 0.91-0.96 across all symptoms. Among these, we observed slightly lower metrics in the visuospatial domain (micro-averaged AUROC: 0.97, 95% CI 0.95-0.99; micro-averaged *F*_1_-score: 0.89, 95% CI 0.85-0.93; micro-averaged precision: 0.87, 95% CI 0.83-0.91; micro-averaged recall: 0.91, 95% CI 0.87-0.94). Table 3 provides a comprehensive evaluation of the performance metrics for both models.

**Table 3:** Performance of the 2-tier hierarchical symptom classification model.

Model		*F*_1_-score^a^, value (95% CI)	AUPRC^a,b^, value (95% CI)	Precision^a^, value (95% CI)	Recall^a^, value (95% CI)	AUROC^a,c^, value (95% CI)	Accuracy^a^, value (95% CI)
Stage I binary symptom classification model		0.98 (0.97-0.98)	1.00 (0.99-1.00)	0.98 (0.97-0.98)	0.98 (0.97-0.98)	1.00 (0.99-1.00)	0.98 (0.97-0.98)
**Stage II multi-label symptom classification model**							
	Memory	0.96 (0.94-0.98)	0.94 (0.91-0.96)	0.96 (0.95-0.98)	0.95 (0.94-0.97)	0.99 (0.98-1.00)	0.94 (0.92-0.96)
	Executive function	0.91 (0.88-0.94)	0.85 (0.82-0.89)	0.90 (0.87-0.92)	0.92 (0.90-0.95)	0.98 (0.97-0.99)	0.87 (0.84-0.90)
	Motor	0.94 (0.92-0.96)	0.90 (0.87-0.92)	0.93 (0.91-0.95)	0.94 (0.92-0.96)	0.98 (0.97-0.99)	0.93 (0.91-0.95)
	Language	0.93 (0.92-0.96)	0.97 (0.97-0.99)	0.93 (0.91-0.96)	0.93 (0.92-0.96)	0.98 (0.96-0.99)	0.91 (0.88-0.94)
	Visuospatial	0.89 (0.85-0.93)	0.82 (0.78-0.87)	0.87 (0.83-0.91)	0.91 (0.87-0.94)	0.97 (0.95-0.99)	0.82 (0.78-0.87)
	Neuropsychiatric	0.91 (0.89-0.95)	0.94 (0.91-0.96)	0.91 (0.88-0.94)	0.92 (0.89-0.94)	0.99 (0.98-1.00)	0.90 (0.87-0.93)
	Sleep	0.96 (0.94-0.98)	0.94 (0.91-0.96)	0.96 (0.94-0.98)	0.96 (0.94-0.98)	0.99 (0.98-1.00)	0.95 (0.92-0.98)

^a^The performance metrics for both models are calculated as micro-averages.

^b^AUPRC: area under the precision-recall curve.

^c^AUROC: area under the receiver operating characteristic curve.

### Model Validation With ADRD Differential Diagnosis

To validate the accuracy of our 2-tier hierarchical symptom classification model, we used a machine learning model to classify ADRD diagnoses with the counts of identified symptoms as model features. We compared 2 L1-regularized logistic regression models: one based on regex-derived symptom counts and another using counts derived from the 2-tier hierarchical LLM. This method allowed us to assess the efficacy of traditional regex techniques against more advanced LLM approaches in the context of ADRD diagnostic accuracy.

**Figure 3:**
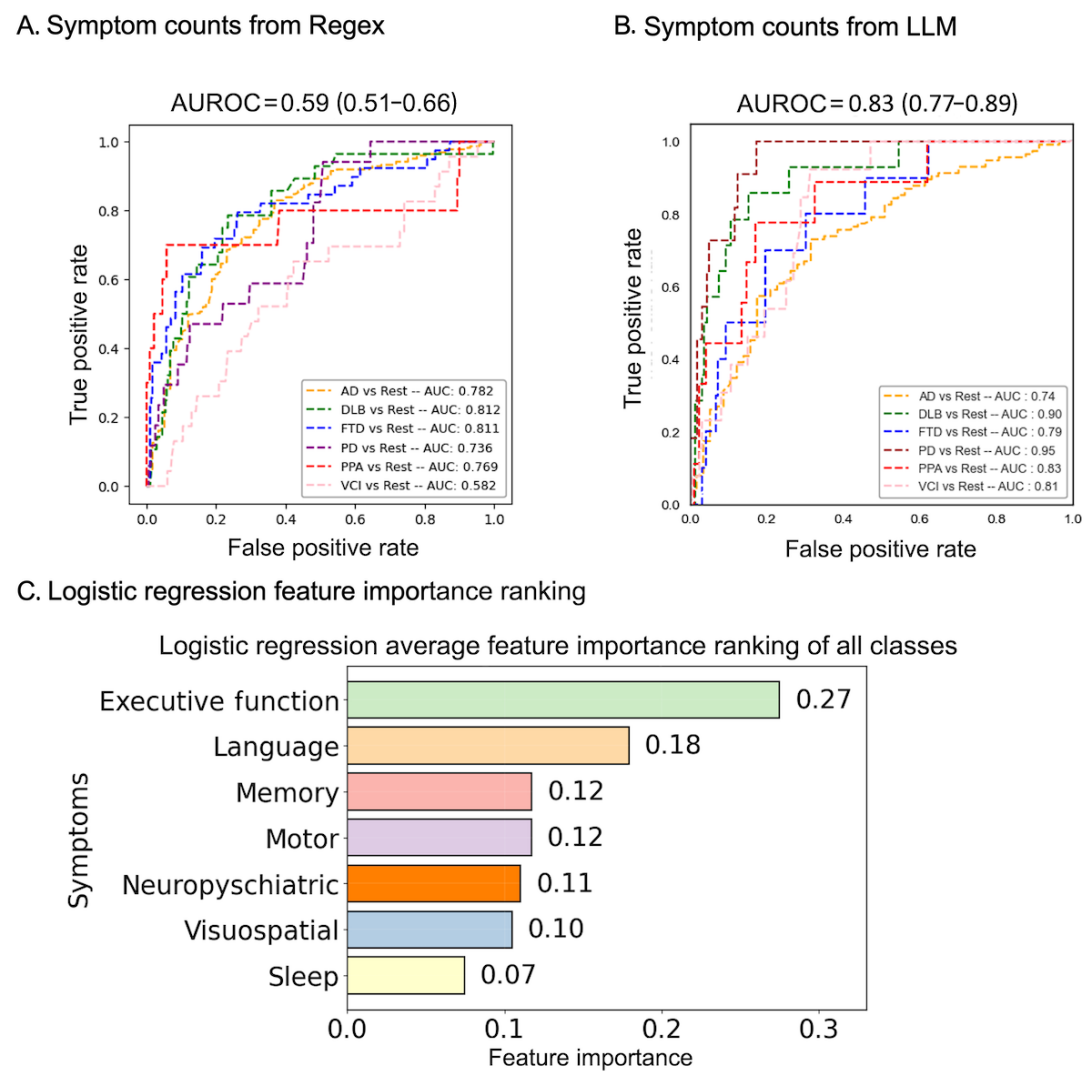
Performance of Alzheimer disease and related dementias (ADRD) differential diagnosis. (A) Receiver operating characteristic (ROC) curves for predicting 6 ADRD diagnoses (Alzheimer disease [AD], dementia with Lewy bodies [DLB], frontotemporal dementia [FTD], Parkinson disease [PD], primary progressive aphasia [PPA], and vascular cognitive impairment [VCI]) using an L1-regularized logistic regression model based on regex-derived symptom counts. The area under the receiver operating characteristic curve (AUROC) is 0.59 (95% CI 0.51-0.66). (B) ROC curves for an L1-regularized logistic regression model using 2-tier hierarchical large language model (LLM)-derived symptom counts. The AUROC is 0.83 (95% CI 0.77-0.89). (C) Feature importance ranking for the model using LLM-derived symptom counts, with an average across the coefficients of symptoms in all ADRD diagnoses. Executive function is the most important feature, followed by language, motor, memory, neuropsychiatric, visuospatial, and sleep. AUC: area under the curve.

First, we predicted ADRD diagnoses using L1 logistic regression based on regex-derived symptom counts. Using regex patterns, we extracted symptom counts from the latest visit notes of 1712 patients diagnosed with ADRD, spanning 7 domains: memory, executive function, motor, language, visuospatial, neuropsychiatric, and sleep. These counts were used to build an L1-regularized multinomial logistic regression model, which predicted the type of ADRD diagnosis using symptom counts as features. The model’s average AUROC was 0.59 (95% CI 0.51-0.66). Detailed AUROC values for each ADRD diagnosis relative to the rest are displayed in Figure 3A.

Second, we predicted ADRD diagnoses using L1 logistic regression based on LLM symptom counts. The second model, leveraging symptom counts extracted from patient visit notes via the 2-tier hierarchical LLM, aimed to predict specific ADRD diagnoses using L1-regularized logistic regression. This model demonstrated a substantial enhancement in diagnostic accuracy, achieving an AUROC of 0.83 (95% CI 0.77-0.89) compared to the AUROC of 0.59 (95% CI 0.51-0.66) obtained with the regex-based model. This marked improvement highlights the model’s efficacy in accurately classifying ADRD categories, underscoring the potential of transformer-based BioBERT models in capturing the context of clinical symptoms from notes. The detailed AUROC for each diagnosis compared to the rest is displayed in Figure 3B.

Further, analysis using feature importance derived from the LLM-based logistic regression model showed that *executive function* had the greatest predictive power on average, followed by *language*, *motor*, *memory*, *neuropsychiatric*, *visuospatial*, and *sleep*. This ranking, illustrated in Figure 3C, emphasizes the critical roles of *executive function*, *language*, *memory*, and *motor* symptoms in predicting ADRD diagnoses. Feature importance rankings for each ADRD diagnosis are illustrated in Figure S3 in Multimedia Appendix 4.

### Model Validation With Brain MRI

**Figure 4:**
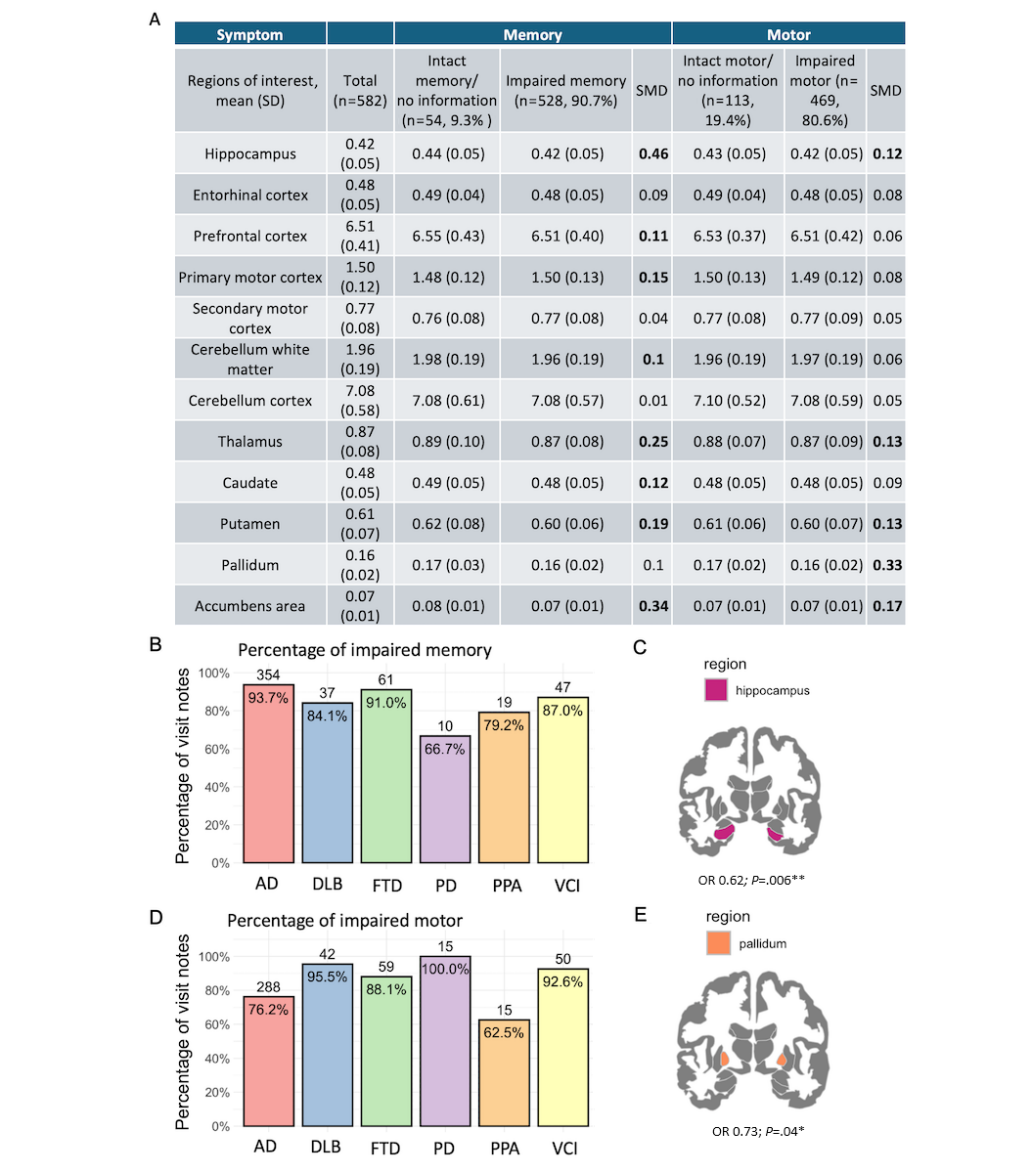
Evaluation of model performance with magnetic resonance imaging brain volume. (A) Summary statistics of the volumes of brain regions associated with memory or motor functions. A standardized mean difference (SMD) threshold of 0.1 has been employed to assess the equilibrium of each metric. Measurements with an SMD exceeding 0.1 (highlighted in bold) signify a comparative lack of balance. (B) Percentage of visit notes with at least one impaired memory symptom predicted by the memory model across visit notes with Alzheimer disease and related dementias (ADRD) diagnosis. The number above each bar represents the number of visit notes in each ADRD diagnosis where impaired memory symptoms were detected. As expected, visit notes with Alzheimer disease (AD) diagnosis had the highest proportion of memory symptoms across all ADRD diagnoses. (C) Coronal view of the brain area associated with memory impairment. Patients with a smaller hippocampus had a higher likelihood of memory impairment (odds ratio [OR] 0.62; *P*=.006). (D) Percentage of visit notes with at least one impaired motor symptom predicted by the motor model across visit notes with ADRD diagnosis. The number above each bar represents the number of visit notes in each ADRD diagnosis where impaired motor symptoms were detected. As expected, visit notes with dementia with Lewy bodies (DLB) and Parkinson disease (PD) diagnoses had the highest proportion of motor symptoms across all ADRD diagnoses. (E) Coronal view of the brain area associated with motor impairment. Patients with a smaller pallidum had a higher likelihood of motor impairment (OR 0.73; *P*=.04). All *P* values have been adjusted for multiple comparisons. FTD: frontotemporal dementia; PPA: primary progressive aphasia; VCI: vascular cognitive impairment. *P<.05, **P<.01.

We used MRI brain volume data to assess our model’s ability to identify symptoms from clinical notes. We hypothesized that the volumes of selected brain regions associated with each domain would be smaller in patients with impaired symptoms predicted from the notes compared to those without. The model analyzed 582 sentences or phrases, identifying memory impairment in 90.7% (528/582) and motor impairment in 80.6% (469/582) of cases. In particular, we observed that memory-impaired individuals showed smaller hippocampal and prefrontal cortex volumes (SMDs >0.1), while motor-impaired individuals had reduced volumes in subcortical regions, including the thalamus, putamen, pallidum, and accumbens area (SMDs >0.1). For brain volume summary statistics from *memory* and *motor* BioBERT model predictions, see Figure 4A.

The *memory* model predicted that visit notes of patients with AD had the highest proportion (93.7%) of *memory* symptoms relative to the other ADRD diagnoses, which is consistent with our understanding that memory impairment is the initial and primary symptom for most patients with AD [2] (Figure 4B). The MRI analysis of *memory* symptoms revealed that a smaller hippocampal volume was associated with an increased likelihood of memory impairment (odds ratio [OR] 0.62, 95% CI 0.46-0.84; *P*=.006) (Figure 4C). Power analysis for the logistic regression, using 1000 simulations, yielded an 89.7% chance of detecting a significant impact of hippocampal volume on *memory* symptoms, thereby confirming the reliability of these findings. Nonetheless, the volumes of the entorhinal cortex and prefrontal cortex did not show a significant relationship with *memory* symptoms (*P*>.05), but the prefrontal cortex had high SMDs (Figure 4A).

In terms of *motor* symptoms, the *motor* model predicted that visit notes with DLB (95.5%) and PD (100%) diagnoses had the highest proportion of *motor* symptoms across visit notes of ADRD diagnoses, which is consistent with our understanding that motor impairment is the primary symptom for patients with DLB and PD [3,5] (Figure 4D). The MRI analysis of *motor* symptoms revealed that a smaller pallidum size was significantly associated with the presence of motor impairments (OR 0.73, 95% CI 0.58-0.9; *P*=.04) (Figure 4E). Power analysis for the logistic regression, conducted with 1000 simulations, revealed an 84.7% probability of accurately detecting a significant influence of pallidum volume on motor symptoms, which substantiates the robustness of our results. Other regions related to motor function did not exhibit significant volumetric differences (*P*>.05). Age and sex were accounted for in all analyses. All results were corrected for multiple comparisons [30]. Thus, the MRI findings corroborated both *memory* and *motor* symptom predictions made by our 2-tier hierarchical LLM.

### Error Analysis

We performed an error analysis to gain insights into the misclassifications made by the 2-tier hierarchical LLM, particularly in its ability to classify symptoms as intact or impaired across the 7 domains. We included both the held-out test set and the MRI validation dataset in our analysis to ensure thoroughness. It is worth mentioning that since the MRI validation dataset does not include true labels, we relied on chart reviews to validate predictions (Figure S1 in Multimedia Appendix 4).

Our error analysis began by examining instances where the models’ predictions of symptoms across ADRD types did not align with known disease profiles. For example, in AD cases, where memory impairment is a prominent symptom [2], the model did not predict *memory* symptoms in 6.1% (23/378) of cases. These instances were notable for their focus on broader cognitive decline or general test scores rather than explicit mentions of *memory* symptoms. In FTD, 93% (62/67) of visit notes referenced *memory* symptoms, which is intriguing since memory impairment is not typical in FTD, particularly in its behavioral variant [31]. Manual review confirmed that these symptoms were indeed documented. In VCI, 87% (47/54) of visit notes mentioned *memory* symptoms, with a consistent recognition of memory issues as a feature of VCI [32]. The model detected *memory* symptoms in 84% (37/44) of DLB visit notes and 79% (19/24) of PPA cases, which often concerned semantic memory challenges. Another example involves *motor* symptoms. The model showed a small margin of error in DLB cases, failing to detect *motor* symptoms in just 2 cases (2/44, 5%). In AD visit notes, *motor* symptoms were predicted accurately in 76.2% (288/378) of notes. FTD cases showed an 88% (59/67) occurrence of *motor* symptoms, and VCI notes included *motor* symptom references in 92.6% (50/54) of cases, often related to lower body motor challenges. PPA patients were identified with *motor* symptoms in 63% (15/24) of notes, with manual verification confirming the presence of true *motor* symptoms in majority (11/15, 73%) of these cases.

The second part of the error analysis investigated visit notes by random sampling, with a focus on notes with high symptom counts (more than 10 symptom predictions). This examination uncovered several types of errors affecting prediction accuracy across all symptoms, including six types of false positives: (1) generalizing cognitive function as a symptom, (2) confusing one symptom with another symptom, (3) identifying evaluation or test statements as impairment, (4) misrecognizing intact as impaired, (5) misleading by ambiguous or complex sentences, and (6) confusing medical history as present symptoms. Four types of false negatives were also identified, including (1) overlooking particular expressions, (2) overlooking particular test scores, (3) misrecognizing impaired as intact, and (4) overlooking sentences or phrases that require contextual information. Table 4 provides a detailed breakdown of these error types and examples from visit notes. Additionally, to understand the distribution of false positives and false negatives across the model’s predictions at the sentence level, we calculated confusion matrices based on the held-out test set for each symptom, and the data are presented in Figure S4 in Multimedia Appendix 4.

**Table 4:** Types of errors in model prediction.

Types of errors			Example (mislabeled category; correct category)
**False positive**			
	Generalizing cognitive function as a symptom	“problem in cognitive functioning” (mislabeled: impaired memory; correct: no information)	
	Confusing one symptom with another symptom	“she began to have trouble sorting items” (mislabeled: impaired memory; correct: impaired executive function) “cannot remember a word” (mislabeled: impaired motor; correct: impaired memory)	
	Identifying evaluation or test statements as impairment	“patient visit for evaluation of memory impairment” (mislabeled: impaired memory; correct: no information)	
	Misrecognizing intact as impaired	“Mild wordfinding difficulty has resolved” (mislabeled: impaired language; correct: intact language) “No disorientation in time” (mislabeled: impaired memory; correct: intact memory) “Plantar response is flexor bilaterally” (mislabeled: impaired motor; correct: intact motor)	
	Misleading by ambiguous or complex sentences	“Speech is fluent but some dysnomia is noted” (mislabeled: intact language; correct: impaired language) “Long term memory is fine but short term memory is not great” (mislabeled: intact memory; correct: impaired memory) “Impairment of short-term memory has declined” (mislabeled: intact memory; correct: impaired memory)	
	Confusing medical history as present symptoms	“ask about his past falls” (mislabeled: impaired motor; correct: no information)	
**False negative**			
	Overlooking particular expressions	“repeat the same question over and over again” (mislabeled: no information; correct: impaired memory) “he puts things away in the wrong place” (mislabeled: no information; correct: impaired memory)	
	Overlooking particular test scores	“CDR-SOB memory is 1” (mislabeled: no information; correct: impaired memory)	
	Misrecognizing impaired as intact	“oriented partially in time” (mislabeled: intact memory; correct: impaired memory) “oriented to his wife but has visual agnosia” (mislabeled: intact visuospatial; correct: impaired visuospatial) “He requires help to dress only for adult undergarments but not for clothes” (mislabeled: intact motor; correct: impaired motor)	
	Overlooking sentences or phrases that require contextual information	“memory has been stable for 2 years. He has worsened in the past 5 months” (mislabeled: intact memory; correct: impaired memory) “Gait: … slow to initiate.” (mislabeled: no information; correct: impaired motor)	

## Discussion

In this study, we developed and evaluated an LLM-based 2-tier hierarchical model for automated symptom extraction, which was trained on expert-labeled visit notes from patients with ADRD at the MGH memory clinic. The model classified sentences or phrases into categories of *impaired*, *intact*, or *no information* for 7 ADRD symptoms: *memory*, *executive function*, *motor*, *language*, *visuospatial*, *neuropsychiatric*, and *sleep*. Our method demonstrated superiority over rule-based and keyword-dependent methods [7-11], which often miss nuanced contextual and semantic relationships. The model achieved robust performance in detecting each symptom from clinical notes, with a micro-averaged AUROC ranging from 0.97 to 0.99. Furthermore, with the implementation of our LLM-based symptom extraction, the AUROC for ADRD differential diagnosis improved substantially (AUROC=0.83) compared to regex-based extraction (AUROC=0.59). Moreover, our model’s predictions aligned with clinical evidence, with most clinical notes correctly matching their respective symptoms. Further, the associations of symptoms with different affected brain regions were substantiated through brain MRI findings. Thus, our model holds potential as a screening tool to streamline diagnosis, improve precision in clinical trials and treatment planning, and enhance our understanding of ADRD subtype heterogeneity.

Traditional approaches, such as regex-based methods, are highly dependent on predefined sets of keywords or rules. They struggle with variations in how symptoms are expressed. For instance, the phrase “difficulty swallowing” could be documented in various ways, such as “unable to swallow” and “has trouble swallowing,” or with more context-specific expressions like “takes 60 minutes to feed the patient a meal.” It is difficult to build a one-size-fits-all rule for captioning every symptom in each domain. To illustrate these challenges, we created a list of regex patterns for ADRD symptoms (Multimedia Appendix 3) and compared the performance of our LLM-based model with traditional regex techniques. We evaluated both methods using 2 L1-regularized logistic regression models: one based on symptom counts derived from regex patterns, and another using counts from our 2-tier hierarchical LLM. Our results showed that the LLM-based model significantly outperformed the regex-based model, achieving an AUROC of 0.83, compared to an AUROC of 0.59 obtained with the regex-based model. This improvement demonstrates the LLM’s ability to better capture the context of clinical symptoms in ADRD, highlighting the superiority of transformer-based models, like BioBERT, in overcoming the limitations of traditional rule-based approaches. Other researchers have used NLP approaches to determine or extract information from clinical notes as well. For example, Prakash et al [33] achieved strong accuracy and *F*_1_-scores (83%-92%) for determining the presence of ADRD severity information in clinical notes using rule-based methods. Similarly, Chen et al [34] developed a rule-based NLP pipeline to extract cognitive test scores and biomarkers from clinical narratives, achieving an *F*_1_-score of 0.9059 across 7 different measures. Their focus was on identifying and harmonizing cognitive test scores in severity categories for patients with ADRD. However, these approaches primarily focus on specific cognitive tests and biomarkers, which are typically more straightforward to identify. In contrast, our method focuses on symptom extraction of sentences across 7 distinct domains. Symptoms are more complex and less structured, requiring a deep understanding of contextual relationships to accurately identify and classify them. Our study verified that the transformer-based BERT model can address this challenge to handle complex medical terminologies and capture the meanings of terms within their context.

As expected, in ADRD differential diagnosis, *memory* emerged as the most crucial symptom for predicting AD (Figure S3A in Multimedia Appendix 4), *motor* was the most significant symptom for predicting DLB (Figure S3B in Multimedia Appendix 4), and *language* was the most important symptom for predicting PPA (Figure S3E in Multimedia Appendix 4). These findings are consistent with our understanding of the clinical manifestations of these diseases [2-4].

While no single disease required all 7 symptoms for prediction (Figure S3 in Multimedia Appendix 4), *executive function* stood out as the most important (for AD, PD, and VCI; see Figures S3A, D, and F in Multimedia Appendix 4) or moderately important (for DLB, FTD, and PPA; see Figures S3B, C, and E in Multimedia Appendix 4) feature across all predictions. Notably, the importance of *executive function* in predicting AD was comparable to that of *memory*. This may be due to the broad range of behaviors associated with *executive function*, such as planning, time management, and working memory, which are intricately woven into the complexity of daily life. Additionally, the frontal lobe, a key hub for *executive function* [35], is extensively connected with other brain regions involved in various functions [36]. For example, *memory* impairment may impact the hippocampal-prefrontal pathway [37], thereby affecting tasks that require both *memory* and *executive function*, such as remembering to take medications at specific times. This pattern also helps explain why, in the case of FTD, a disease characterized by severe behavioral manifestations [4] and frontal or temporal lobe degeneration [38], *executive function* provides only moderate predictive power. Although this might seem counterintuitive given the role of *executive function* in FTD, it may be because the behavioral symptoms in FTD are more prominent, and *executive function* may not have sufficient discriminatory power for a differential diagnosis. Moreover, frontal lobe atrophy in FTD may affect behavior in a manner similar to how disruption in the connection between the frontal lobe and other functional areas impacts executive tasks, thereby influencing the overall predictive value of *executive function* in this context.

In the context of ADRD differential diagnosis, our model identified *memory* as a moderately important symptom on average for diagnosing ADRD (Figure 3C). When evaluating prediction performance by specific ADRD diagnoses, *memory* was ranked as the most crucial symptom for predicting AD (Figure S3A in Multimedia Appendix 4); moderately important for FTD (Figure S3C in Multimedia Appendix 4) and VCI (Figure S3F in Multimedia Appendix 4); and least important for DLB (Figure S3B in Multimedia Appendix 4), PD (Figure S3D in Multimedia Appendix 4), and PPA (Figure S3E in Multimedia Appendix 4). This importance ranking for *memory* aligns with existing knowledge about the prevalence of memory impairment across different ADRD diagnoses [2-5,32]. The model generally performed well in identifying *memory* symptoms. However, in some patients with AD, *memory* symptoms were not predicted. Further analysis revealed that this was likely due to follow-up notes simply stating “no change” in the patient’s condition, which did not trigger the model’s detection mechanisms. This suggests a need for improvement in detecting implied or static memory impairments. Additionally, some notes detailed atypical AD presentations, emphasizing language or motor difficulties rather than memory loss, which can indicate variations in clinical presentation among patients with the same underlying etiology. Further, an unexpectedly high prevalence of *memory* symptoms in FTD underscores the complexity of symptomatology. While aging has been suggested as a confounding factor for *memory* symptoms in FTD [4], our data indicated no significant age difference in patients with and without *memory* symptoms. Meanwhile, some studies have suggested that *memory* symptoms can emerge in patients with progressive FTD, akin to AD presentations [39], which may explain our observation. In DLB cases, our model detected *memory* symptoms in many visit notes, with only 1 case later reclassified as AD. Although DLB typically lacks early memory impairment, such symptoms can develop as the condition advances [3]. Most evaluated visit notes were from initial visits, suggesting that DLB diagnoses might already be at more advanced stages by then. Further analysis showed that AD cases had more frequent memory-related references than DLB (Wilcoxon rank sum test W=105474; *P*<.001), demonstrating our model’s ability to distinguish patterns of the same symptom across different diagnoses.

Motor symptoms were the most prevalent impairments among patients with ADRD in our dataset (Table 2) and showed moderate importance on average in predicting ADRD diagnoses (Figure 3C). When evaluating prediction performance by specific ADRD diagnoses, *motor* was ranked as the most crucial symptom for predicting DLB (Figure S3B in Multimedia Appendix 4); moderately important for AD (Figure S3A in Multimedia Appendix 4), PD (Figure S3D in Multimedia Appendix 4), PPA (Figure S3E in Multimedia Appendix 4), and VCI (Figure S3F in Multimedia Appendix 4); and least important for FTD (Figure S3C in Multimedia Appendix 4). This importance ranking for *motor* aligns with existing knowledge about the prevalence of motor impairment in AD, DLB, PPA, and VCI diagnoses [2-4,32]. The low ranking of *motor* in predicting FTD and its moderate ranking for PD was unexpected, considering that these 2 diseases have more behavioral symptoms closely associated with motor function [4,5]. This discrepancy might be due to the broad range of *motor* functions involved, making it harder to distinguish nuances between these diseases and others, similar to the case where *executive function* had a moderate contribution in predicting FTD. As expected, patients with DLB or PD had the highest occurrences of *motor* symptoms. Notably, 1 patient initially diagnosed with mild cognitive impairment was later found to have DLB, which the model had correctly predicted, underscoring the model’s robustness. FTD cases often exhibited *motor* symptoms, even though their diagnoses did not change to DLB or PD in later visits. This was observed despite excluding *motor* symptom subtypes like corticobasal syndrome or progressive supranuclear palsy [4], and no motor neuron diseases were noted. This underscores that motor symptoms can develop in patients with FTD over time, even when they are not diagnosed with conditions typically associated with these symptoms. Moreover, patients with FTD having *motor* symptoms were generally older, aligning with symptom progression, although the age difference was not statistically significant. In patients with AD, the model’s prediction of frequent *motor* symptoms, such as “unsteady stance” and “perseveration of movement” (largely confirmed upon chart review), aligns with literature indicating that late-stage AD can manifest motor impairments [2], similar to those seen in DLB or PD [3,5]. This suggests that these patients with AD may be at more advanced stages of the disease. Patients with AD having *motor* symptoms were generally older, which is consistent with the progression hypothesis, though this relationship was not statistically significant. The high occurrence of *motor* symptoms in VCI cases (confirmed through manual review), which emphasized sentences or phrases that particularly mention the lower body being affected, such as “gait instability” and “frequent falls,” aligns with clinical knowledge [32]. Only 1 predicted VCI case was later diagnosed with DLB, highlighting the model’s specificity for differential diagnosis.

Among all symptom predictions, *visuospatial* symptoms had the lowest performance (Table 3). Further review revealed that certain behaviors might reflect mixed symptoms in patients’ clinical presentations. For example, “unable to drive” in clinical notes could be due to impaired navigation ability [40-42], typically categorized as a *visuospatial* symptom, but driving is a complex behavior that also involves *executive function* for planning the route [43], *memory* for remembering place names [43], and *motor* skills for physical control [43]. Therefore, developing more refined models that can better distinguish and specifically target *visuospatial* symptoms will be essential for improving the accuracy of symptom extraction.

This study has several limitations. While our current NLP techniques proved to be effective in symptom extraction, the model performance is still susceptible to diverse clinical narratives and abbreviations. For example, we tailored data preprocessing templates for each provider, which makes it challenging to generalize the model to different health care settings. Additionally, our study focused on patients with a single ADRD diagnosis, yet many patients fall into the dementia unspecified category due to mixed dementia. For instance, autopsy studies revealed that patients with pure VCI were less common than those with mixed dementia [44], which often co-occurred with AD pathology [45] and complicated the diagnostic process. Finally, our method is primarily intended for research use, and several challenges, such as data privacy, clinician–artificial intelligence interaction, and model performance, need to be overcome before it is ready for clinical decision-making.

Future studies should include patients with multiple ADRD diagnoses and at different disease stages to better reflect real-world complexities. Enhancements might include more sophisticated language parsing and the integration of clinical criteria for improved specificity. Moreover, integrating structured patient data, such as demographics and neurological tests, could enhance the model’s precision and generalizability. Recent studies, such as the study by Xue et al [46], have shown the potential of transformer-based models for multi-modal differential diagnosis of dementia, suggesting avenues for further refinement of our approach. Furthermore, the dataset generated through our efforts provides a foundation for successive cycles of the active learning loop, having the potential to continually refine and elevate the model’s performance over time. Future research should leverage this dataset to further improve model performance and explore avenues for expanding the scope of symptom extraction in diverse clinical scenarios.

## Appendix

Multimedia Appendix 1 Alzheimer disease and related dementias diagnosis list.

Multimedia Appendix 2 Symptom domain and examples.

Multimedia Appendix 3 Alzheimer disease and related dementias symptom regular expression list.

Multimedia Appendix 4 Supplementary data to support the findings.

## Abbreviations

AD: Alzheimer disease
ADRD: Alzheimer disease and related dementias
AUROC: area under the receiver operating characteristic curve
DLB: dementia with Lewy bodies
EHR: electronic health record
FTD: frontotemporal dementia
LLM: large language model
MGH: Massachusetts General Hospital
MRI: magnetic resonance imaging
NLP: natural language processing
PD: Parkinson disease
PPA: primary progressive aphasia
SMD: standardized mean difference
VCI: vascular cognitive impairment
