# Supplementary material for: High-Throughput Phenotyping of the Symptoms of Alzheimer Disease and Related Dementias Using Large Language Models: Cross-Sectional Study
Source: JMIR AI. 2025 Jun 3;4:e66926. doi: 10.2196/66926 (PMC12174885; doi:10.2196/66926)
Supplement: Multimedia Appendix 4 [file ai_v4i1e66926_app4.docx]

**Multimedia Appendix 4.** Supplementary data to support the findings.


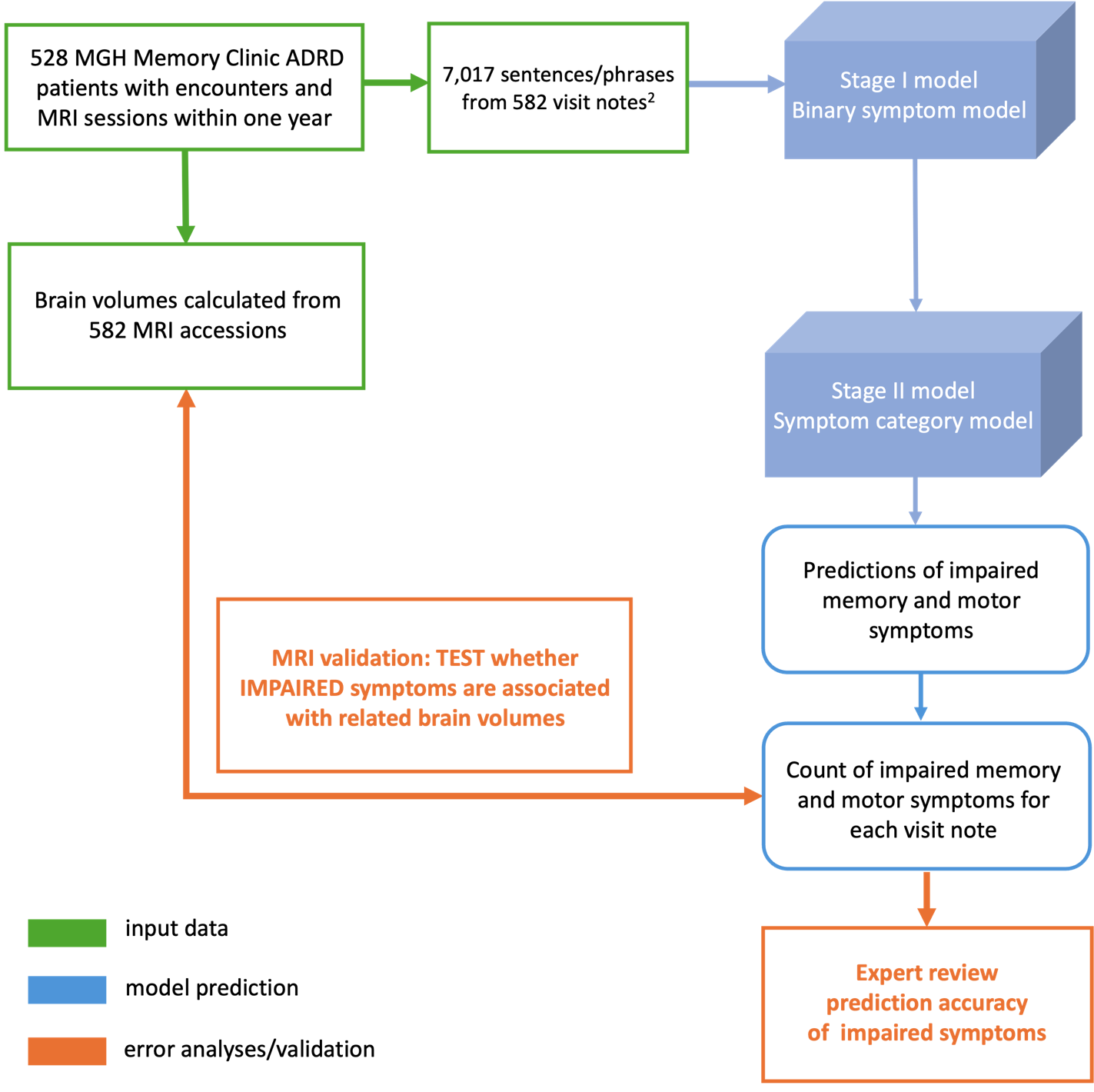


**Figure S1.** Overview of the MRI validation workflow. A schematic representation of the procedure for symptom prediction using the MRI validation dataset, the subsequent corroboration of these predictions with the MRI data, and the analysis of any discrepancies encountered.

**
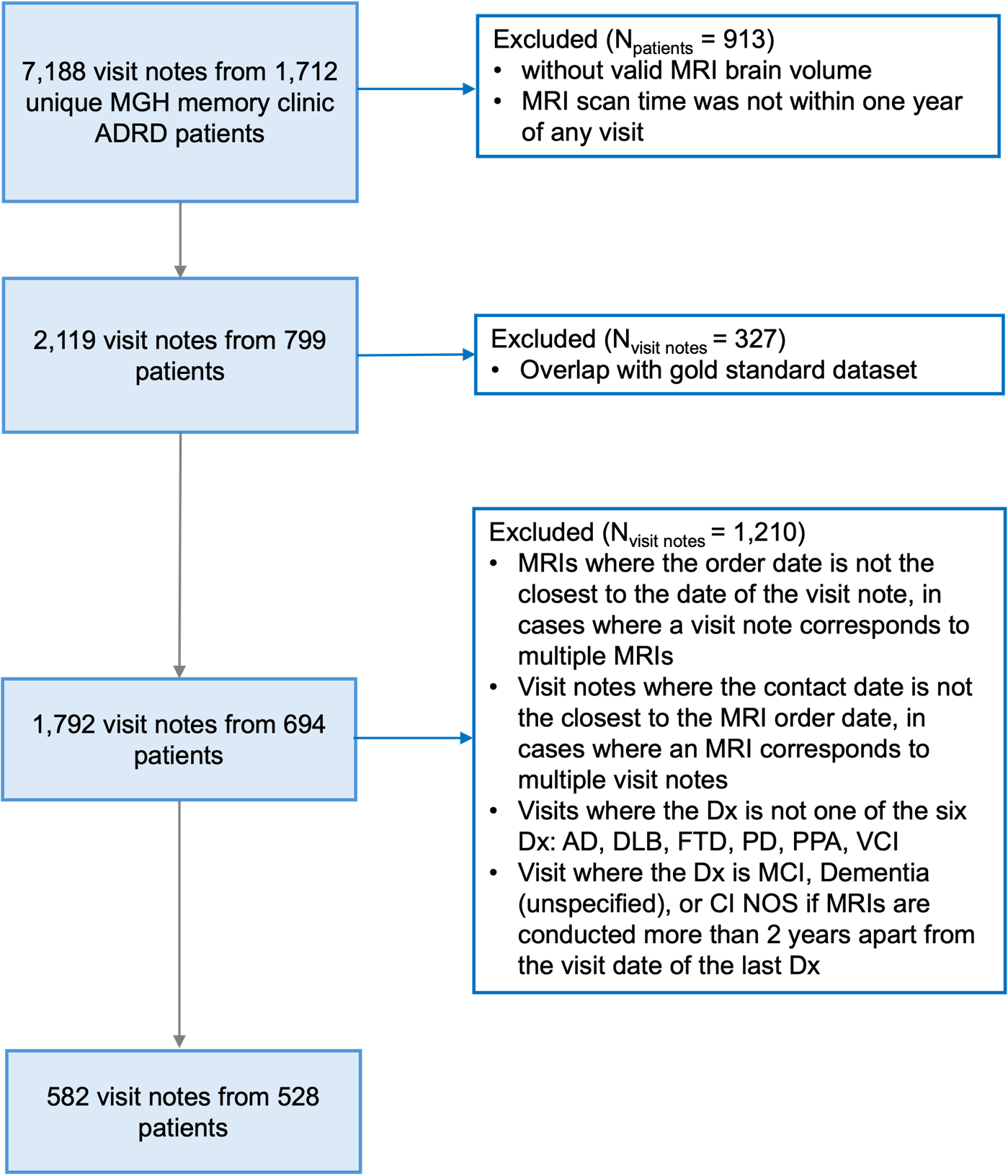
**

**Figure S2.** Consort diagram illustrating the extraction of the MRI validation dataset from patient records at the MGH memory clinic.


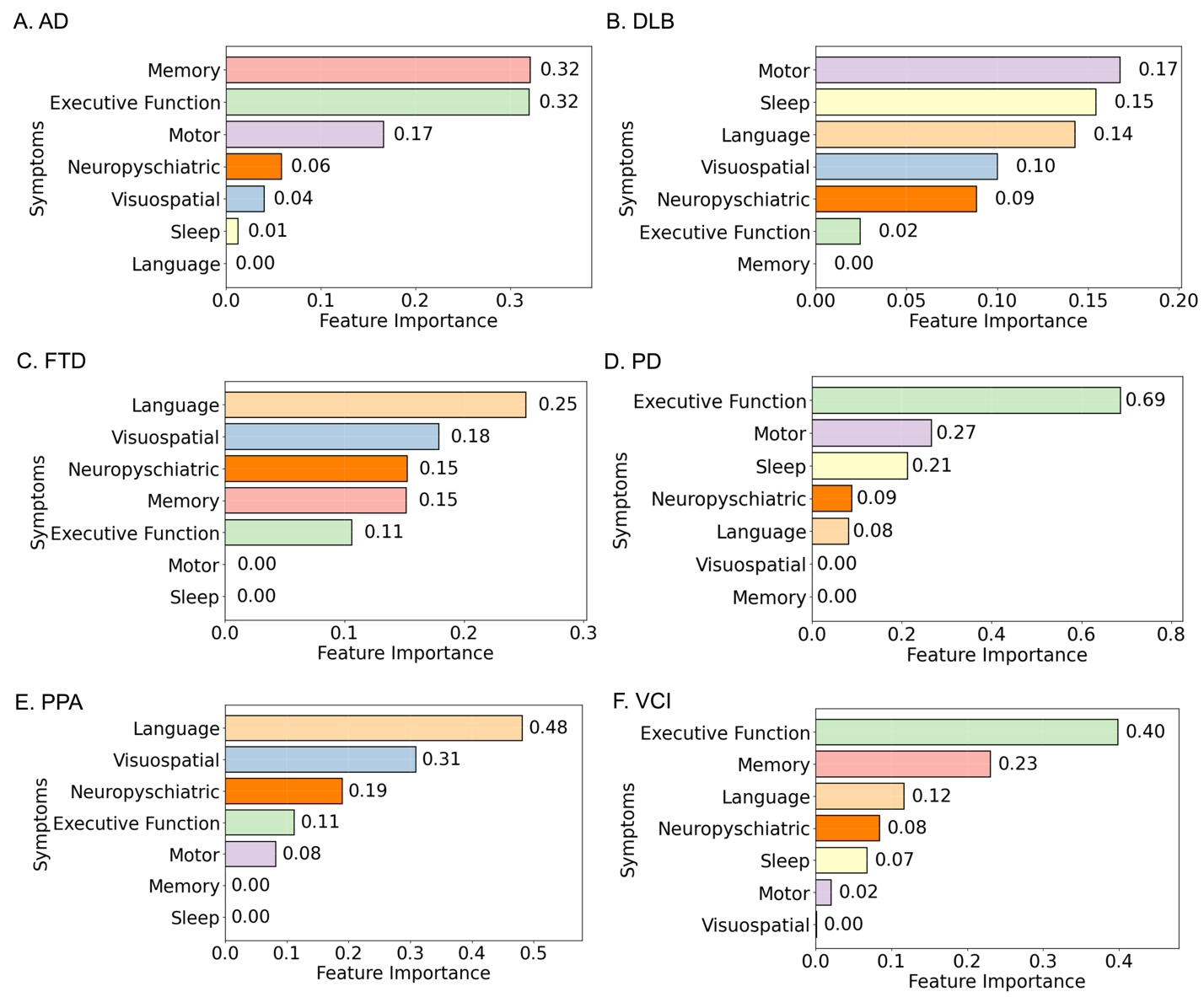


**Figure S3.** Feature importance ranking for the model using LLM-derived symptom counts, separate by ADRD diagnoses. A) Feature importance ranking in Alzheimer’s disease (AD) prediction, where Memory and Executive Function were equally listed as the most important feature; B) Feature importance ranking in dementia with Lewy bodies (DLB) prediction where Motor was the most important feature; C) Feature importance ranking in frontotemporal dementia (FTD) prediction where Language was the most important feature; D) Feature importance ranking in Parkinson’s disease (PD) prediction where Executive Function was the most important feature; E) Feature importance ranking in primary progressive aphasia (PPA) prediction where Language was the most important feature; F) Feature importance ranking in vascular cognitive impairment (VCI) prediction where Executive Function was the most important feature.

*
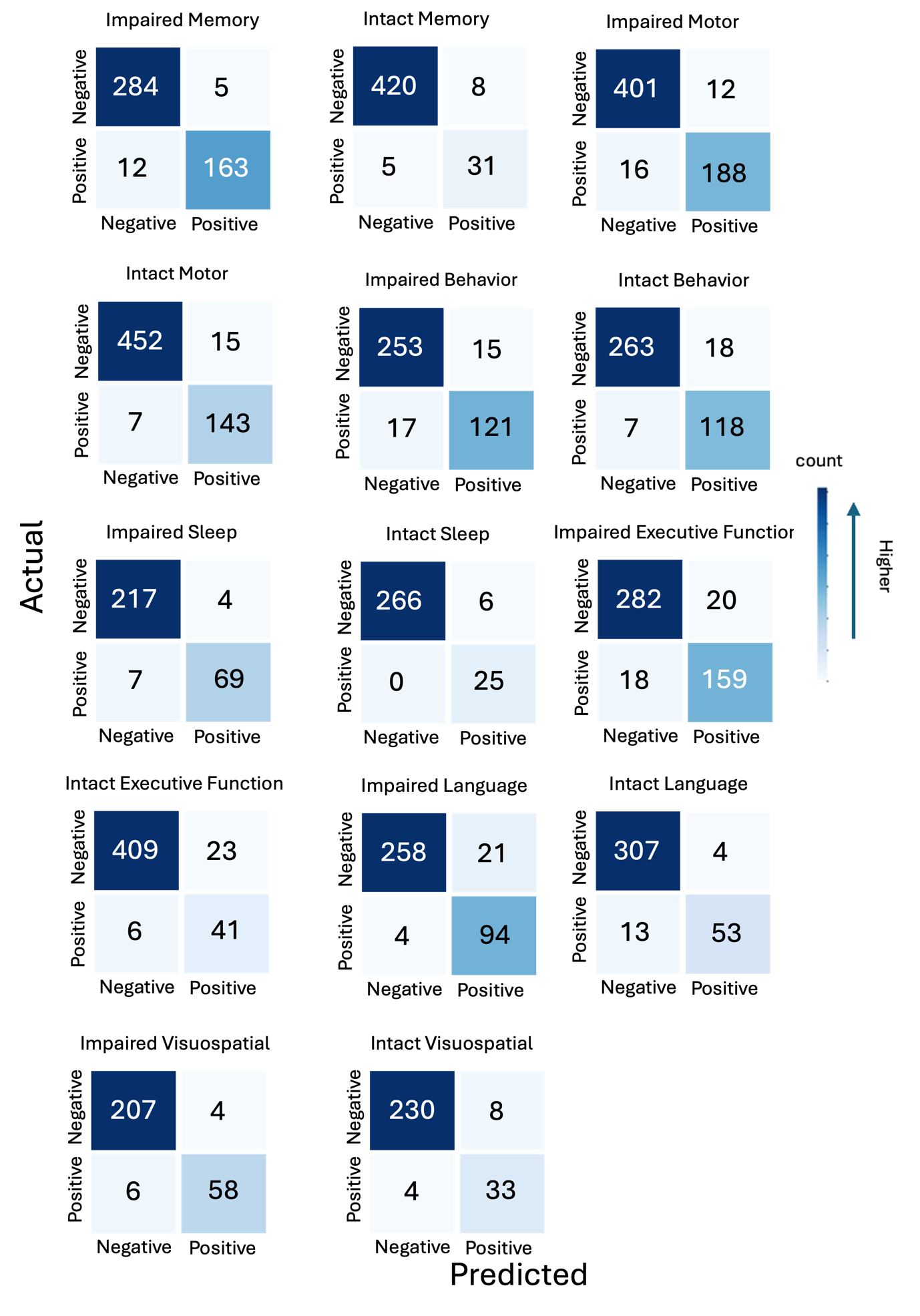
*

**Figure S4.** Confusion matrices of the two-tier hierarchical symptom classification model for each ADRD symptom category based on the hold-out test set . Going by order: Confusion Matrices Impaired Memory and Intact Memory, Confusion Matrices Impaired Motor and Intact Motor, Confusion Matrices Impaired Behavior and Intact Behavior, Confusion Matrices Impaired Sleep and Intact Sleep, Confusion Matrices Impaired Executive Function and Intact Executive Function, Confusion Matrices Impaired Language and Intact Language, Confusion Matrices Impaired Visuospatial and Intact Visuospatial. Note: we did not combine intact and impaired results into the same confusion matrix because some sentences/phrases have both impaired and intact symptoms within the same sentence.
